# ‘Sugarcoating’ 2-deoxyglucose: mechanisms that suppress its toxic effects

**DOI:** 10.1007/s00294-020-01122-7

**Published:** 2020-11-02

**Authors:** Martin C. Schmidt, Allyson F. O’Donnell

**Affiliations:** 1 Department of Microbiology and Molecular Genetics, University of Pittsburgh School of Medicine, Pittsburgh, PA 15219 USA; 2 Department of Biological Sciences, University of Pittsburgh, Pittsburgh, PA 15260 USA

**Keywords:** AMP-activated protein kinase, SNF1, α-Arrestin, Hexose transporter, PP1 phosphatase

## Abstract

Yeast and cancer cells are metabolically similar as they use fermentation of glucose as a primary means of generating energy. Reliance on glucose fermentation makes both of these cell types highly sensitive to the toxic glucose analog, 2-deoxyglucose. Here we review the cellular and metabolic pathways that play a role in 2-deoxyglucose sensitivity and discuss how the modifications to these pathways result in acquisition of 2-deoxyglucose resistance. Insights gained from genetic and proteomic studies in yeast provide new ideas for the design of combinatorial therapies for cancer treatment.

## The impact of 2-deoxyglucose on metabolism

**Fig. 1:**
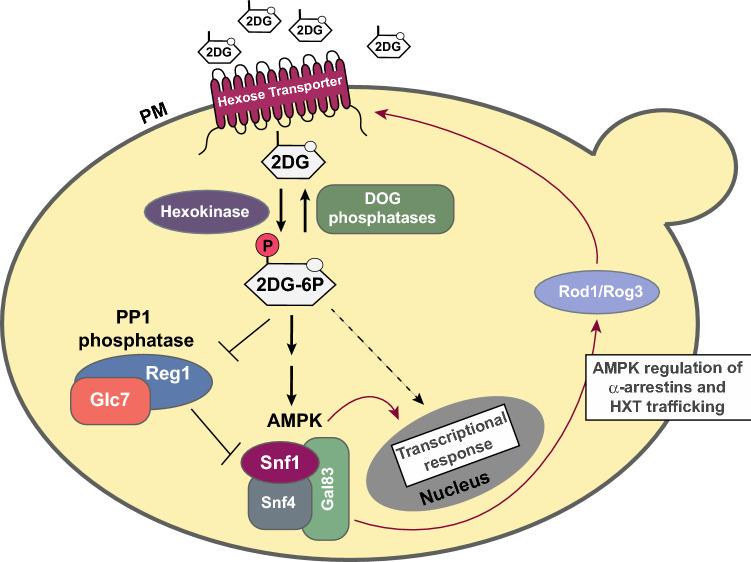
Model for 2DG uptake and signaling. 2DG is taken up by yeast cells by hexose transporter (HXT) proteins and phosphorylated to 2DG-6P by hexokinase enzymes. Accumulation of 2DG-6P is toxic to cells. Cellular adaptation to the presence of 2DG requires the Snf1/AMPK kinase complex. Resistance to 2DG can be imparted by activation of Snf1 kinase signaling via dominant alleles in the genes encoding the kinase subunits or by loss-of-function alleles in the genes (*GLC7* and *REG1*) encoding the PP1 phosphatase that down-regulates Snf1. Adaptation to 2DG is also promoted by mutations in the hexokinase 2 gene (*HXK2*) or up-regulation of the *DOG1* and *DOG2* phosphatases, which reduce production or enhance the degradation of the toxic 2DG-6P, respectively. Nearly all mutations that confer 2DG resistance increase HXT retention at the cell surface, which is controlled in a Snf1- and α-arrestin-dependent manner in response to 2DG. While Snf1 regulation of Mig1 (not show) alleviates repression of some genes in response to 2DG, other transcriptional responses to 2DG likely to occur via Snf1-independent signaling pathways

Glucose is the preferred energy source for the majority of cells. Glucose enters cells through the glucose transporters, named GLUTs in mammalian cells, which are widely conserved and orthologous to hexose transporters (HXTs) in the budding yeast *Saccharomyces cerevisiae* (Ozcan and Johnston 1999). Upon uptake, glucose is metabolized through glycolysis and oxidative phosphorylation to produce adenosine triphosphate (ATP), which provides the energy for most cellular functions. Glucose metabolism is derailed by addition of 2-deoxyglucose (2DG), a glucose analog that lacks the hydroxyl group on carbon 2 in the glucose backbone. Just like glucose, 2DG is taken up by glucose transporters, however once inside the cell, 2DG wreaks havoc on the normal metabolic rhythm by perturbing glycolytic flux; 2DG is converted to 2-deoxyglucose-6-phosphate (2DG-6P) by hexokinase, but 2DG-6P cannot be used by phosphoglucose isomerase in the next enzymatic step of glycolysis and it accumulates as a metabolic dead end. Accumulation of 2DG-6P leads to inhibition of hexokinase itself (Chen and Guéron 1992), hampering the metabolism of glucose and creating starvation-like conditions in the cells as ATP levels plummet. Recent studies reveal a more complex impact of 2DG on cellular physiology, and here we review these new advances in our understanding of how cells evade the toxic effects of 2DG (Fig. 1). A more complete understanding of how cells become resistant to 2DG will aid in the development of novel, combinatorial anti-cancer therapeutics, as cancerous cells are particularly susceptible to 2DG since they are glucose addicted and heavily dependent upon glycolysis.

## 2DG as a cancer therapeutic

Interest in understanding the mechanism of action for 2DG has been renewed by this drug’s use in clinical trials as an anti-cancer therapeutic in combination with other drugs (Maher et al. 2004; Maschek et al. 2004; Pajak et al. 2019). Central to the use of 2DG as an anti-cancer treatment is the fact that cancer cells often undergo a dramatic metabolic shift—first identified by Otto Warburg nearly 100 years ago (Warburg et al. 1927)—whereby even in the presence of oxygen these cells forgo oxidative phosphorylation in favor of fermenting glucose to lactic acid in a process known as ‘aerobic glycolysis’. To accommodate this new metabolic lifestyle, cancer cells become dependent upon high levels of glucose uptake to support glycolysis as the primary mode of ATP production. These features make cancer cells particularly susceptible to 2DG as they take up more 2DG than surrounding healthy cells and are more vulnerable to the 2DG-induced inhibition of glycolysis and subsequent drop in ATP levels. The elevated influx of 2DG into cancerous cells has long been exploited as a diagnostic tool to identify and image cancers in patients by monitoring the preferential uptake of a 2DG-related compound, ^18^F-2-deoxyglucose, via positron emission tomography scans (Pajak et al. 2019). While the use of 2DG as an anti-cancer therapeutic is promising, studies of the potency of 2DG revealed that frequent resistance to 2DG developed in cancerous cells as well as many other cell types (Barban 1961; Heredia and Sols 1964; Maher et al. 2007; Zhong et al. 2009). A more complete understanding of how cells respond to 2DG and the routes commonly used to evade the toxicity of this compound will not only advance our views of these fundamental metabolic pathways but will also facilitate rational drug design and development of improved combinatorial therapeutics that prevent cancer cell resistance during treatment.

## Yeast as a model to understand the cellular impact of 2DG

The metabolic similarities between the budding yeast *Saccharomyces cerevisiae* and cancerous cells that have undergone the Warburg shift make yeast an attractive model system for studying cellular responses to 2DG. However, all of the early studies of 2DG in yeast utilized cultures growing by fermentation of alternative sugars (sucrose, raffinose, galactose), a metabolic condition not directly applicable to mammalian tumor cells. The early yeast studies identified genes whose products participate in control of glucose repression, including hexokinase II, protein phosphatase 1 (PP1), as well Snf1, the yeast ortholog of the mammalian AMP-activated protein kinase (AMPK) (Entian and Zimmermann 1980; Neigeborn and Carlson 1987; Zimmermann et al. 1977; Zimmermann and Scheel 1977). More recent studies in yeast have focused on defining mechanisms of 2DG-resistance in cells growing on glucose, which is more germane to what a cancerous cell would experience (Defenouillere et al. 2019; McCartney et al. 2014; O'Donnell et al. 2015; Ralser et al. 2008; Soncini et al. 2020).

**Fig. 2:**
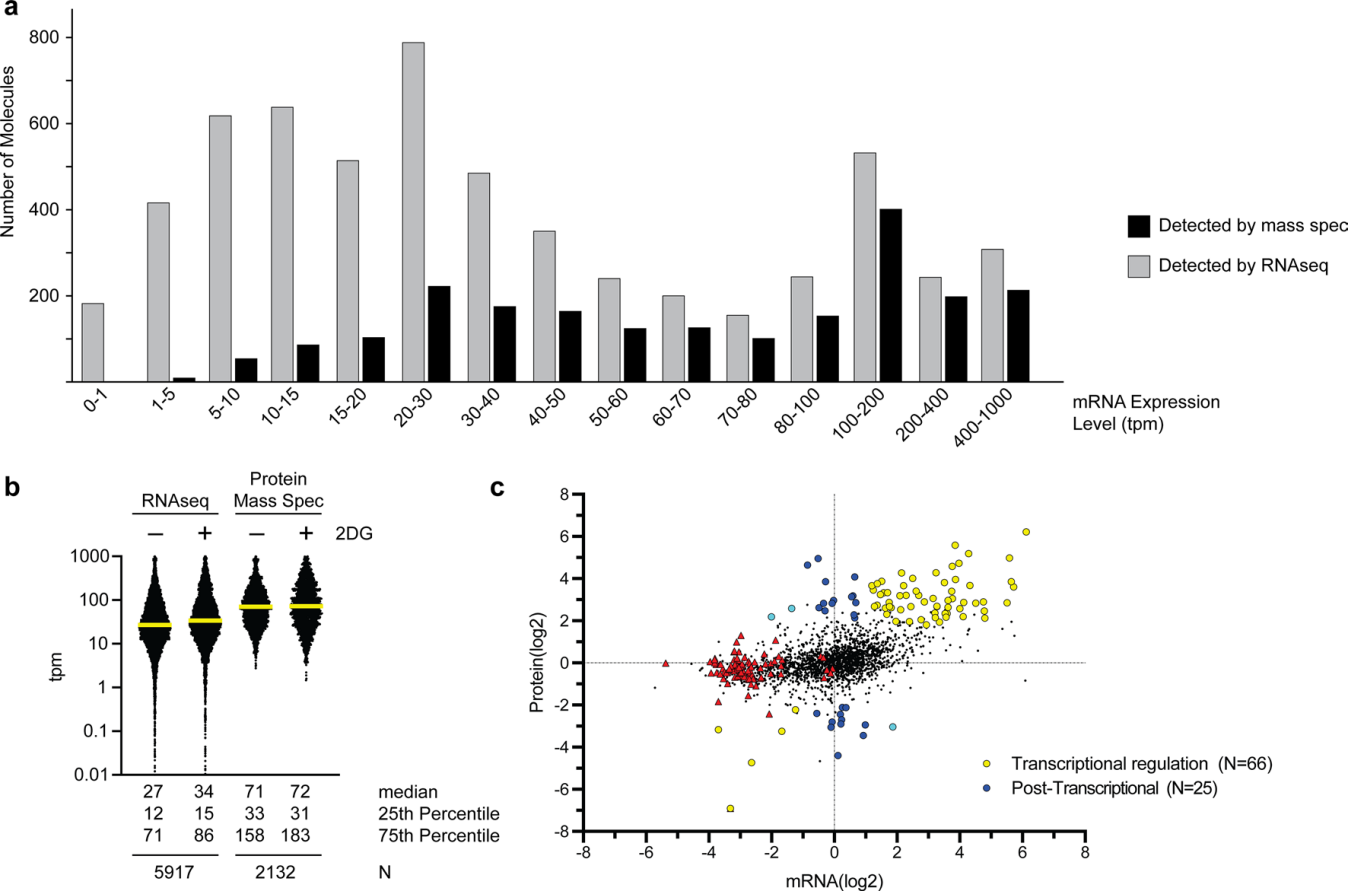
Comparison of 2DG response measured by RNAseq and mass spectrometry. **a** The number of molecules (mRNA or protein) detected by RNAseq and mass spectrometry are plotted as a function of mRNA abundance (transcripts per million mapped reads; tpm). **b** Abundance of mRNA is plotted for all the detected molecules with the median value shown in yellow and a summary of distribution for each column shown below. **c** Changes in the abundance of mRNA and protein following exposure to 2DG. Data for all of the 2132 proteins detected by mass spectrometry are shown in circles (smaller black dots or larger colored dots). Those which surpassed the mass spectrometry false discovery rate threshold (FDR < 0.01) are shown as larger, colored circles. Values are plotted as the log2 ratio of the level detected in 2DG over the level detected in the absence of 2DG. Transcriptionally regulated genes (defined as showing > twofold change in both mRNA as measured by RNAseq and protein levels as measured by MS) are shown as yellow circles. Post-transcriptionally regulated proteins (defined as showing > twofold change in protein with < twofold change in mRNA) are shown as dark blue circles and proteins where the protein results from MS were anti-correlated with the RNAseq transcriptional data are shown as light blue dots. Ribosomal proteins are shown as red triangles for comparison

Comparison of the glucose-grown yeast cell response to 2DG at the mRNA level as measured by RNAseq (Soncini et al. 2020) and at the protein level as measured by mass spectrometry (Defenouillere et al. 2019) reveals that these methods are complementary, each with its own strengths and weaknesses (Fig. 2). RNAseq is more sensitive as it is able to detect mRNA for almost all 6000 of the yeast genes. In contrast, mass spectrometry detects proteins from just over one third of the 6000 yeast genes, primarily those with more abundant mRNA (Fig. 2a, b). Of the 94 proteins whose changes in abundance satisfy a stringent false discovery rate (FDR < 0.01), most (70%) also show corresponding changes in mRNA abundance suggesting that transcriptional regulation underlies much of the 2DG response detected by proteomics (Fig. 2c). The transcription of the ribosomal protein genes is drastically reduced following 2DG exposure (Soncini et al. 2020) yet the abundance of these stable proteins does not change appreciably over the short time courses of these experiments (2–3 h). Additionally, comparison of these two datasets suggests that the abundance of a smaller class of proteins is regulated post-transcriptionally since their mRNA abundances do not change in a commensurate manner with the changes in their protein abundance. The mechanism(s) by which 2DG mediates the post-transcriptional regulation of protein abundance remain to be determined. These two datasets combined yield a comprehensive view of the yeast cell’s response to 2DG. The most significant enzymes and pathways that give rise to 2DG resistance are reviewed below.

## Mechanisms of 2DG resistance in yeast

Recent studies have identified genes and signaling pathways that can confer 2DG resistance to yeast cells growing on glucose. Ralser and colleagues screened the haploid yeast knockout collection for cells that acquire 2DG resistance (Ralser et al. 2008). While most of the genes identified in this screen did not reproducibly generate 2DG resistance using more rigorous genetic analyses (McCartney et al. 2014), this study was the first to demonstrate the importance of analyzing 2DG resistance on glucose grown cells. An unbiased screen for spontaneous mutants that confer 2DG resistance to glucose grown cells (Soncini et al. 2020) identified mutations hexokinase II, AMPK and subunits of the PP1 phosphatase, a known regulator of AMPK. The observation that cells lacking AMPK are hypersensitive to 2DG (McCartney et al. 2014) and that cells with hyperactive AMPK are resistant (McCartney et al. 2014; Soncini et al. 2020) underscores the importance of AMPK in the response and adaptation to life in the presence of 2DG. In a complementary approach, the Leon lab used proteomics to define the cellular response to 2DG and identified overlapping as well as additional pathways that confer 2DG resistance (Defenouillere et al. 2019). This proteomics-based study underscored the importance of up-regulation of the two DOG phosphatases (Dog1 and Dog2), enzymes that confer resistance through dephosphorylation of the toxic metabolite 2-deoxyglucose-6-phosphate (2DG-6P). The most significant enzymes and pathways that give rise to 2DG resistance and the mechanism of 2DG toxicity are outlined in our model (Fig. 1) and reviewed below.

## Hexose transporters: the double-edged swords of 2DG toxicity

**Fig. 3:**
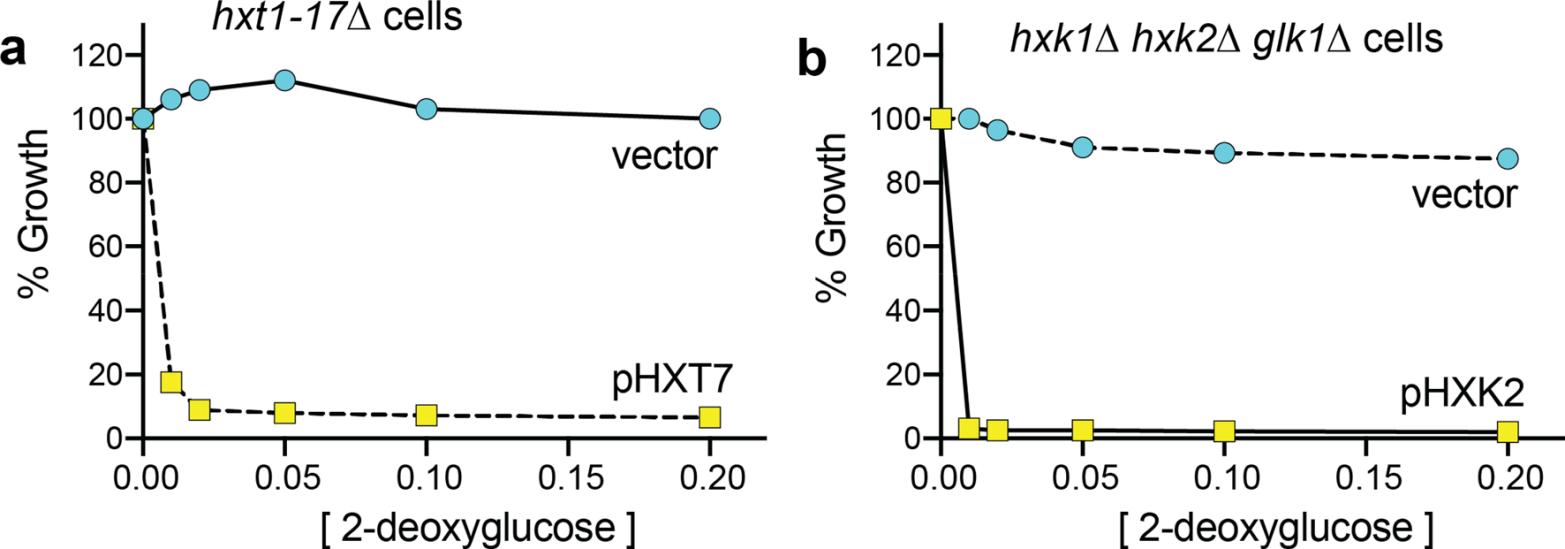
Toxicity of 2DG requires uptake and phosphorylation. **a** Uptake of 2DG through the HXT membrane transporters is necessary for 2DG toxicity. Cells lacking all 17 *HXT* genes (Roy et al. 2014) can grow on the disaccharide maltose and are completely resistant to even high concentrations of 2DG. Introduction of a single *HXT* gene on a plasmid restores sensitivity to 2DG. **b** Phosphorylation of 2DG is necessary for 2DG toxicity. Cells lacking all three hexokinase genes can grow on galactose and are resistant to even high concentrations of 2DG. Introduction of the *HXK2* gene on a plasmid restores 2DG toxicity

2DG toxicity requires that it first be taken into the cell via the hexose transporters. Deletion of all 17 of the yeast hexose transporters as is the case in *hxt1-17∆* cells (Roy et al. 2014), results in complete resistance to 2DG (Fig. 3a) with the presence of a single *HXT* transporter sufficient to restore 2DG toxicity. Paradoxically, however, in cells exposed to 2DG that are glucose grown, retention of the glucose transporters at the cell surface aids in cellular resistance to 2DG (O'Donnell et al. 2015). The presence of 2DG creates a dilemma for the cell, whereby it needs to retain glucose transporters at the cell surface to take up glucose for fermentation and synthesis of ATP, but the same transporters also allow for the toxic 2DG to enter the cell and disrupt normal cellular metabolism. Almost all of the 2DG-resistant mutants identified in our screen increased retention of Hxt3 at the cell surface (Soncini et al. 2020) and the severe 2DG sensitivity of *snf1*∆ cells can be restored to near wild-type levels by over-expression of glucose transporters Hxt1 or Hxt3 (O'Donnell et al. 2015), both of which demonstrate the importance of continued glucose internalization in mitigating 2DG toxicity. Key mediators of glucose transporter abundance at the cell surface in response to 2DG are the α-arrestins, a class of protein trafficking adaptor. Their role in regulating HXTs in this context is described in more detail below.

## Hexokinase II and the production of a ‘dead-end’ metabolite from 2DG

Once internalized, 2DG is converted to 2DG-6P by the action of hexokinase. Yeast express three hexokinases—Hxk1, Hxk2 and Glk1—and two of these—Hxk1 and Hxk2—are able to efficiently phosphorylate both glucose and 2DG to create glucose-6-phosophate or 2DG-6P, respectively (Soncini et al. 2020). In the absence of these three enzymes, as is the case in *hxk1*∆ *hxk2*∆ *glk1*∆ yeast strain, cells can no longer grow by fermentation of glucose and they become completely resistant to 2DG (Fig. 3b). Thus, both entry of 2DG and its conversion to 2DG-6P are needed for toxicity.

Screens for mutations that confer resistance to 2DG have identified many loss-of-function alleles in hexokinase II (Defenouillere et al. 2019; Lane et al. 2018; McCartney et al. 2014; Soncini et al. 2020; Zimmermann and Scheel 1977). Interestingly, while there are three hexokinases in yeast, only mutations in Hxk2 confer 2DG resistance in these screens (Soncini et al. 2020) and it will be interesting to uncover why these other hexokinases are seemingly unable to contribute to the resistance pathway, especially in light of the fact that hexokinase Glk1 mRNA and protein abundance are both highly elevated in response to 2DG (Defenouillere et al. 2019; Soncini et al. 2020). The Hxk2 enzyme appears to have multiple functions in the cell including influencing Snf1 activation (McCartney et al. 2014), translocating to the nucleus (Fernandez-Garcia et al. 2012) and altering transcription of genes (Fernandez-Garcia et al. 2012; Rodríguez et al. 2001). These and many other proposed alternative functions of Hxk2 (Amigoni et al. 2013; Fernandez-Garcia et al. 2012; Herrero et al. 1989, 1998; Hohmann et al. 1999; Yao et al. 2015) may contribute to its unique role in 2DG resistance. A common feature of the *HXK2* mutations that confer 2DG resistance is that they result in higher levels of phosphorylated, and presumably activated, Snf1 kinase (McCartney et al. 2014; Soncini et al. 2020). It will be interesting to further explore the nuanced role of these Hxk2 mutants in conferring resistance to 2DG by altering cellular signaling and gene expression landscapes.

## A reversal of fortune: the role of the DOG phosphatases in alleviating 2DG sensitivity

Intracellular accumulation of 2DG-6P is thought to be a primary cause of 2DG toxicity (Chen and Guéron 1992). Accumulation of 2DG-6P can be mitigated by the action of two 2DG-6P phosphatases, called Dog1 and Dog2 in yeast, that can dephosphorylate 2DG-6P. Over-expression of either of the *DOG* genes (Randez-Gil et al. 1995), or conditions that lead to increased expression of these enzymes (Defenouillere et al. 2019), confers resistance to 2DG. *DOG1* and *DOG2* genes are adjacent and closely related genes on chromosome 8 that encode phosphatase enzymes in the halo-acid dehalogenase (HAD) family. Yeast express at least 20 proteins with PFAM domains described as “haloacid dehalogenase-like hydrolase”. Most of the HAD phosphatases act on small molecular weight compounds, although four are known to act on phospho-proteins (Offley and Schmidt 2019). The best characterized HAD enzymes are the glycerol phosphate phosphatases Gpp1 and Gpp2 that generate glycerol in response to osmotic stress (Pahlman et al. 2001). The cognate substrate for the DOG phosphatases in vivo is not known although the *DOG* genes are also induced in response to osmotic stress (Defenouillere et al. 2019). The catalytic activity of the DOG phosphatases acting on 2DG-6P has been measured (Randez-Gil et al. 1995) and the Km values are relatively high (17 and 41 mM for Dog1 and Dog2, respectively) suggesting that cells exposed to 2DG accumulate extremely high intracellular concentrations of 2DG-6P. Multiple stress response signaling pathways including the osmolarity stress Hog1 pathway, the metabolic stress Snf1 pathway, the unfolded protein response pathway and the cell wall integrity pathway can up-regulate expression of the *DOG* phosphatases to promote resistance to 2DG (Defenouillere et al. 2019). This same mechanism of 2DG resistance has been observed in human cells where overexpression of a human HAD phosphatase in HeLa cells increases resistance to 2DG (Defenouillere et al. 2019).

## Activation of the AMP-activated protein kinase, Snf1

As 2DG-6P is produced in the cell, it accumulates and impairs normal metabolism of glucose. Metabolic stress and reduced energy status activate the yeast AMPK, Snf1. Key substrates of Snf1 in this context include the Mig transcriptional repressors and the α-arrestins Rod1 and Rog3 (O'Donnell et al. 2015). The Mig1 and Mig2 transcriptional repressors are regulated by multiple stress response signaling pathways (Shashkova et al. 2015; Westholm et al. 2008) and in response to low glucose conditions, their phosphorylation by Snf1 leads to increased expression of a suite of genes (Westholm et al. 2008). In response to 2DG, phosphorylation of Mig1 has not been readily observed (McCartney et al. 2014), however there is a drop in the abundance of Mig1 and RNAseq and MS analyses show that a suite of Mig1-regulated genes are altered in response to 2DG, including increased expression of the *DOG* genes (Defenouillere et al. 2019; Soncini et al. 2020). In addition to regulating transcriptional changes, Snf1-mediated phosphorylation of another substrate, the α-arrestins Rod1 and Rog3, serves to limit endocytosis of the hexose transporters (O'Donnell et al. 2015) thus preserving glucose uptake and metabolism. While α-arrestins were not identified in our screen for mutants that confer resistance to 2DG, consistent with the idea that most mutations that confer resistance to 2DG alter Snf1 activity in some way, we found that all but one of our 2DG-resistant mutants increased glucose transporter abundances at the cell surface (Soncini et al. 2020). Mutations affecting the Snf1 kinase pathway include activating mutations in the *SNF1* gene (McCartney et al. 2014; Soncini et al. 2020) and loss of function alleles in the PP1 phosphatase, a negative regulator of Snf1 (Sanz et al. 2000; Tu and Carlson 1994). Interestingly, screens for 2DG resistance mutants discovered loss of function mutations in either Glc7 or Reg1, subunits of PP1, that give rise to constitutive activation of Snf1 and thereby confer resistance to 2DG (McCartney et al. 2014; Neigeborn and Carlson 1987; Soncini et al. 2020). The function of Reg1 in 2DG-resistance is entirely dependent upon the Snf1 kinase (McCartney et al. 2014). While Snf1’s regulation of the Mig1 repressor and the α-arrestins each contribute to Snf1-mediated resistance to 2DG, they are not the only elements that operate downstream of Snf1. A dominant allele of *SNF1* can promote 2DG resistance in cells lacking the genes for these targets, indicating that additional 2DG-associated Snf1 regulatory functions remain to be discovered (Soncini et al. 2020).

## Prospects for improving the toxic punch of 2DG

Taken together, the data from these recent studies in yeast and the earlier foundational work in this model system create a holistic view of cellular acquisition of 2DG resistance. The Snf1-mediated regulation of glucose transporter localization and abundance, and transcriptional repression of genes plays a major role in regulating cellular response to 2DG. These roles are conserved from yeast to humans; AMPK regulates the α-arrestin TXNIP to control trafficking of the mammalian glucose transporter GLUT1 in response to 2DG (Wu et al. 2013) and mammalian AMPK signaling regulates transcription (Hardie 2011), though the transcriptional response to 2DG in mammalian cells has yet to be directly measured. The conservation of these enzymes and pathways makes the work done in yeast highly applicable to studies of 2DG resistance in mammals. Since activation of Snf1 is such a robust mechanism for side-stepping the toxic effects of 2DG in yeast, perhaps combinatorial therapies in mammals should include not just 2DG, but also AMPK inhibitors. The idea of inhibiting AMPK as a part of cancer treatment may at first appear counterintuitive. Many studies have reported that activation of AMPK is linked to a reduced risk of cancer (Evans et al. 2005; Wang and Guan 2009; Yung et al. 2013) and AMPK activation as part of combinatorial treatments for cancer may prove to be especially effective for those cancers that inactivate tumor suppressors like LKB1 (a kinase that phosphorylates AMPK to activate it) and TSC2 (a direct substrate of AMPK that when phosphorylated impedes TORC1 activation when phosphorylated) (Chen et al. 2017; Hardie and Alessi 2013; Inoki et al. 2006; Lizcano et al. 2004). However, the use of AMPK inhibitors in applications where 2DG is present may help prevent cancer cells from spontaneously becoming resistant to 2DG. Certainly, for anti-cancer therapies where 2DG is used, the addition of AMPK activators, which would likely render 2DG ineffective, seems a poor idea. Instead, the implementation of Compound C, which reversibly inhibits AMPK but may also have ‘off-target’ effects, or the indirect inhibition of AMPK with the fatty acid synthesis inhibitor C75—each of which have been used as neuroprotective measures after stroke (Viollet et al. 2010)—might be considered as additions to regimens that contain 2DG. Based on the genetic screens completed in yeast to date, the extensive conservation of this pathway in mammalian cells, and the high rate of spontaneous resistance to 2DG-toxicity already observed in many cell types secondary inhibition of Snf1/AMPK should make 2DG a more potent inhibitor of cell growth, helping it to pack a more ‘toxic’ punch. In our opinion, this avenue of adding AMPK inhibitors to 2DG-containing anti-cancer cocktails to help guard against spontaneous suppression is worthy of further consideration for combinatorial cancer treatments.
